# Hospital performance comparison of inpatient fall rates; the impact of risk adjusting for patient-related factors: a multicentre cross-sectional survey

**DOI:** 10.1186/s12913-022-07638-7

**Published:** 2022-02-18

**Authors:** Niklaus S Bernet, Irma HJ Everink, Jos MGA Schols, Ruud JG Halfens, Dirk Richter, Sabine Hahn

**Affiliations:** 1 School of Health Professions, Applied Research & Development in Nursing, Bern University of Applied Sciences, Murtenstrasse 10, 3008 Bern, Switzerland; 2 Department of Health Services Research, Care and Public Health Research Institute, Maastricht University, PO BOX 616, MD 6200 Maastricht, The Netherlands; 3 Center for Psychiatric Rehabilitation, Bern University Hospital for Mental Health, Murtenstrasse 46, 3008 Bern, Switzerland; 4 University Hospital for Psychiatry and Psychotherapy, University of Bern, Bolligenstrasse 111, 3060 Bern, Switzerland

**Keywords:** Accidental Falls, Benchmarking, Cross-Sectional Studies, Hospitals, Inpatients, Quality Improvement, Risk Adjustment, Risk Factors

## Abstract

**Background:**

Comparing inpatient fall rates can serve as a benchmark for quality improvement. To improve the comparability of performance between hospitals, adjustments for patient-related fall risk factors that are not modifiable by care are recommended. Thereafter, the remaining variability in risk-adjusted fall rates can be attributed to differences in quality of care provided by a hospital. Research on risk-adjusted fall rates and their impact on hospital comparisons is currently sparse. Therefore, the aims of this study were to develop an inpatient fall risk adjustment model based on patient-related fall risk factors, and to analyse the impact of applying this model on comparisons of inpatient fall rates in acute care hospitals in Switzerland.

**Methods:**

Data on inpatient falls in Swiss acute care hospitals were collected on one day in 2017, 2018 and 2019, as part of an annual multicentre cross-sectional survey. After excluding maternity and outpatient wards, all inpatients older than 18 years were included. Two-level logistic regression models were used to construct unadjusted and risk-adjusted caterpillar plots to compare inter-hospital variability in inpatient fall rates.

**Results:**

One hundred thirty eight hospitals and 35,998 patients were included in the analysis. Risk adjustment showed that the following factors were associated with a higher risk of falling: increasing care dependency (to a great extent care dependent, *odds ratio* 3.43, *95% confidence interval* 2.78–4.23), a fall in the last 12 months (*OR* 2.14, *CI* 1.89–2.42), the intake of sedative and or psychotropic medications (*OR* 1.74, *CI* 1.54–1.98), mental and behavioural disorders (*OR* 1.55, *CI* 1.36–1.77) and higher age (*OR* 1.01, *CI* 1.01–1.02). With odds ratios between 1.26 and 0.67, eight further ICD-10 diagnosis groups were included. Female sex (*OR* 0.78, *CI* 0.70–0.88) and postoperative patients (*OR* 0.83, *CI* 0.73–0.95) were associated with a lower risk of falling. Unadjusted caterpillar plots identified 20 low- and 3 high-performing hospitals. After risk adjustment, 2 low-performing hospitals remained.

**Conclusions:**

Risk adjustment of inpatient fall rates could reduce misclassification of hospital performance and enables a fairer basis for decision-making and quality improvement measures. Patient-related fall risk factors such as care dependency, history of falls and cognitive impairment should be routinely assessed.

**Supplementary Information:**

The online version contains supplementary material available at 10.1186/s12913-022-07638-7.

## Introduction

Inpatient falls in hospitals and subsequent injuries are a widely recognized and highly relevant health problem associated with lower quality of life, longer hospital stays and higher healthcare costs [1–3]. A fall is defined as “any unintentional change in position that results in the client coming to rest on the ground or other lower level, regardless of the reason” [4]. For inpatients in acute care hospitals falls are one of the most frequently reported safety accidents [5–7]. Falls thus generate a high amount of additional costs, as shown for example by data from the UK. More than 2.7% of the 7.4 million people admitted to acute care hospitals in the UK in 2015/2016 experienced a fall incident, which, converted into international dollars according to the Organisation for Economic Co-operation and Development (OECD) [8], led to total annual costs for UK acute care hospitals of around $739 million [7].

Inpatient falls are considered to be a nursing-sensitive quality of care indicator, as they are healthcare-acquired, mostly preventable and, as described, have serious consequences for patients, hospitals and the health care system [3, 9]. Accordingly, measuring and comparing fall rates can serve as a benchmark for quality improvement in hospitals when one hospital’s performance is compared with that of other hospitals, but also for accountability purposes such as public reporting [10]. A prerequisite for a meaningful comparison is that there is a potential for improvement. This is indicated if the hospitals report different fall rates, i.e., there is a certain degree of variability across the hospitals [11]. We therefore searched the literature for observational studies reporting hospital-level inpatient fall rates based on large sample sizes. The inpatient fall rates found range from 1 to 17% [12–16]. The identified variability in inpatient fall rates across hospitals could be, in addition to random chance, explained by the following three factors [17]. First, differences in the definition of fall events and data quality related to different data collection methods and the documentation of fall events can significantly influence inpatient fall rates and therefore limit comparability between hospitals [3]. Second, the variability may be due to the fact that hospitals’ performance in preventing inpatient falls, and thus the clinical quality of care, varies considerably. Third, variability may also be explained by differences in patient-related fall risk factors in the hospitals [10]. These patient-related fall risk factors are specific conditions that increase a person’s chance of falling but are mainly beyond the control of hospitals [10, 11, 18].

According to the Registered Nurses’ Association of Ontario (RNAO) [19], over 400 fall risk factors have been described. However, there are only a limited number of general, well-researched patient-related fall risk factors such as advanced age, history of falls, cognitive impairment, the use of psychotropic medication and impaired gait, balance and or mobility [19, 20]. In addition, there are also inconsistent findings: for example, to what extent male sex represents a fall risk factor [20–22]. Furthermore, for other potential patient-related fall risk factors such as comorbidity or diabetes, no information could be provided due to a limited number of available study results or non-comparable operationalisations of the risk factors [20]. In this context, it is not surprising that no universally applicable fall risk model is available, which is also reflected in the fact that the most commonly used standardised fall risk screening tools rely on different fall risk factors to assess at-risk patients [23–25].

Nevertheless, in order to enable a fair comparison of hospital performance, especially when comparing on the national level and including different hospital types, the presence of patient-related fall risk factors in patient populations must be considered, as patients are not randomly allocated to hospitals and can therefore vary considerably from hospital to hospital [26]. Otherwise, hospitals treating patients with a disproportionate share of patient-related fall risk factors may be affected by higher fall rates and therefore lower hospital performance, even if they work with the highest safety standards [10, 11]. Risk adjustment (also known as case-mix adjustment) is therefore generally recommended to facilitate a meaningful and fair comparison of performance between hospitals [26, 27]. Risk adjustment attempts to control for patient-related risk factors that cannot be influenced by care, so that the remaining variability in risk-adjusted fall rates can be attributed with some certainty to differences in the quality of care provided by hospitals. Accordingly variables related to care processes or structures are not included in risk adjustment models [10].

Unfortunately, little has been published on risk adjustment in relation to falls. Our search in PubMed in February 2021, using the Medical Subject Headings (MESH) term “Risk Adjustment”, which was introduced in 1999, led to 3,644 hits. When it was entered in combination with the MESH terms “Accidental Falls” and “Hospitals”, the search results dwindled to one hit. This article describes the importance of risk adjustment in quality comparisons [28]. An additional search on CINAHL with the same search terms yielded no further relevant results. While several articles describe or use the method of risk adjustment in relation to health care outcomes, e.g., hospital mortality, readmission or surgical procedures, to the best of our knowledge there have been no risk-adjusted fall rates published for acute care hospitals. However, this would appear to be imperative if hospitals do not want to be compared “only” on the basis of unadjusted (crude) fall rates, especially since an unadjusted hospital comparison may lead to inaccurate conclusions about hospital performance, as Danek, Earnest [18] have shown in the field of diabetes care.

Therefore, the aim of this study was, firstly, to develop and describe an inpatient fall risk adjustment model based on patient-related fall risk factors, and secondly, to analyse the impact of applying this model to a comparison of inpatient fall rates of acute care hospitals in Switzerland.

## Methods

### Study design

Data on inpatient falls in acute care hospitals in Switzerland were collected in November 2017, 2018 and 2019 as part of an annual multicentre cross-sectional survey, coordinated by Maastricht University (the Netherlands), titled National Prevalence Measurement of Quality of Care (in Dutch: Landelijke Prevalentiemeting Zorgkwaliteit [LPZ]).

The LPZ measurement takes place in Switzerland, the Netherlands, Austria, UK and Turkey in the hospital, nursing home and home care setting and offers the opportunity to collect data on various quality of care indicators such as inpatient falls, pressure ulcers and malnutrition [29]. In this study, only data on inpatient falls in Swiss acute care hospitals were included in the analysis.

### Setting and sample

In Switzerland, all acute care hospitals that have joined the national quality contract (approximately 97% of Swiss acute care hospitals) participated in the survey. Except for the maternity and outpatient wards, all ward types were included in the measurement. On the day of the measurement, all inpatients older than 18 years for whom informed consent had been given personally or by their legal representative were included [30].

### Measures

For data collection, the LPZ instrument in its revised version (LPZ 2.0) was used [29]. It contains three questionnaires related to three levels: an institutional, a ward and a patient questionnaire.

The institutional and ward questionnaires provide general information on the type of hospital/ward as well as structure and process measures. The patient questionnaire is divided into two parts. A general part in which basic patient data are collected and an indicator-specific part, in which data on the respective quality of care indicator are collected; in our study these were data on falls.

In the present study, information on the type of hospital (university hospital, general hospital or specialised clinic) was taken from the institutional questionnaire. The following variables were used from the general part of the patient questionnaire: age in years, sex, surgical procedure within 14 days prior to measurement day (no/yes), the 21 medical diagnosis groups of the ICD-10 (International Statistical Classification of Diseases and Related Health Problems 10th Revision) [31], each of which was answered with yes or no, and care dependency. Care dependency was measured by the Care Dependency Scale (CDS) [32]. The scale consists of 15 categories (e.g., food and drink, continence, mobility), which are assessed based on five response categories (completely dependent to completely independent). The sum score ranges from 15 to 75 points, where a lower value represents more care dependency [33, 34]. The sum score can be divided into the following categories: 15–24 (completely dependent on care from others), 25–44 (to a great extent dependent), 45–59 (partially dependent), 60–69 (to a great extent independent) and 70–75 (almost care independent) [35].

From the fall indicator-specific part of the patient questionnaire, three out of five questions were relevant for this study: Intake of sedative/psychotropic medications (yes/no), fall history, measured with the question “has the client fallen in the 12 months before hospital admission?” (yes/no) and the outcome variable (inpatient falls), measured retrospectively with the question “has the client fallen in the last 30 days in this institution?” (yes/no). To ensure that the information is available on the day of the measurement, nurses are required to document all falls during the 30 days prior to the measurement (Fachhochschule B: Messhandbuch Schweiz - Nationale Prävalenzmessung Sturz und Dekubitus 2019 im Rahmen der Internationalen Prävalenzmessung von Pflegequalität, LPZ International, Unpublished). The definition of a fall, on which the measurement is based, is described in the introduction section.

The LPZ instrument in its basic version was psychometrically tested, particularly with regard to the quality of care indicator pressure ulcers, and was assessed as being reliable and valid [36–38]. The indicator fall is based on expert opinions and thus achieves face validity [38]. The content and questions of the LPZ instrument are based on evidence-based research and are evaluated annually by the international research group and adapted if necessary [30].

### Data collection

Prior to measurement, national coordinators organized instruction meetings for hospital coordinators to provide training on all relevant aspects of the survey such as using the questionnaires and the data entry program [30].

For reliability purposes, the hospital coordinators define clinical measurement teams consisting of two nurses. One of the nurses works on the ward in question and the other works in a different ward [29]. The measurement teams were trained by the hospital coordinators on how to collect data at patient level using the patient questionnaire. This questionnaire indicates which questions must be answered by clinical examination or questioning of the patient and which questions can be answered using data from medical records.

To ensure uniform data collection, all information about measurement, such as definitions, instructions for completing the questionnaires and technical aids were summarized in a manual (Fachhochschule B. Messhandbuch Schweiz - Nationale Prävalenzmessung Sturz und Dekubitus 2019 im Rahmen der Internationalen Prävalenzmessung von Pflegequalität, LPZ International, Unpublished), which was available to the hospital coordinators and the measurement teams.

The data gathered were entered into the web-based data entry program on the LPZ website, which could only be completed after all mandatory questions had been answered in order to avoid missing values. The data collection for the present study took place on Tuesday, November 14, 2017, Tuesday, November 13, 2018 and Tuesday, November 12, 2019.

### Data analysis

First, the individual data sets from the 2017, 2018 and 2019 measurements were merged into one data set using IBM© SPSS© Statistics (version 27). The cases from the three measurement time points were assigned to the respective hospitals so that an overall fall rate could be calculated for each hospital over the three measurement time points and the number of cases per hospital could be increased for the development of the risk adjustment model. Very small hospitals with a total of less than 50 participants over the 3 measurement years were excluded from the analysis. This is in accordance with simulation studies suggesting a minimum of 50 participants per cluster to estimate accurately within a multilevel logistic modelling approach [39, 40].

Second, the sample was described by using numbers, percentages, 95% confidence interval (95% *CI*), median and interquartile range (IQR).

Third, an unadjusted multilevel logistic regression model (null-model or intercept-only model), which solely models the variability between hospitals regarding inpatient falls by using random intercepts, was calculated. The approach of multilevel logistic regression was chosen to account for the hierarchical structure of the data (patients grouped in hospitals) [41]. The null-model served afterwards as a reference model in three respects: (1) to assess the outcome heterogeneity between hospitals measured by the Intraclass Correlation Coefficient (ICC) [42]; (2) to compare the model fit of the subsequent risk-adjusted model; (3) to visualize the unadjusted hospital performance in a caterpillar plot and, therefore, to detect low- and high-performing hospital outliers if no risk adjustment was undertaken.

Fourth, as a starting point for selecting the relevant patient-related fall risk factors to incorporate in the risk adjustment model, a (non-hierarchical) binary logistic regression model (full model) incorporating all variables described in the measures section was calculated. The continuous variable age was grand-mean centred because it is not reasonable to estimate an age of 0 in our sample, and to avoid convergence problems [40]. Next, based on the full model, the patient-related fall risk factors to adjust for were determined by using a stepwise backward selection algorithm with the Akaike Information Criterion (AIC) [43, 44]. The exploratory approach was chosen to obtain a reduced model from the multitude of possible patient-related fall risk factors, which is limited to the most central risk factors. In the context of risk-adjusted hospital comparison, reduced models are easier to communicate, reduce the effort spent on data collection and usually have the same predictive power as full models without exerting a clinical effect on the hospital comparison [45, 46]. The AIC criterion is suitable for this by penalising more complex models and therefore reducing overfitting [47].

Fifth, an initial risk-adjusted multilevel logistic regression model (risk-adjusted model) was developed that incorporates the patient-related fall risk factors found in step four by using fixed effects, and the grouping variable hospital as a random effect. To test for a possible measurement year effect, we recalculated the initial risk-adjusted model by including the measurement year as a control variable. The measurement year was not significant in the model and the AIC value was higher than in the initial risk adjusted model. Therefore, the initial risk adjusted model was subsequently reported. For the analysis of the variability of the hospital effects we extracted the residuals of the hospitals and their 95% confidence intervals from the fitted models by using the method proposed by Rabe-Hesketh and Skrondal [48] and plotted them in a ranked order in a caterpillar plot. The null model was compared with the risk-adjusted model by using AIC as well as marginal and conditional R^2^ fit indices according to Nakagawa and Schielzeth [49] and the likelihood ratio test. In all analyses the statistical significance level was set at *p* < 0.05.

The statistics software R, version 3.6.3 [50] with the packages *mass* [51], *lme4* [52] *ggplot2* [53] and *sjplot* [54] were used to select the risk adjustment variables as well as to fit and plot the models.

#### Ethical considerations

For the first measurement in 2011, Full Research Ethics Committee approval was granted by the Ethics Committee of the Canton of Bern on 4 October 2011 (application no. 122/11). In accordance with Swiss legislation for national multicentre studies, the other twelve local ethics committees also gave their approval. From the second measurement in 2012 onwards, on the recommendation of the Ethics Committee of the Canton of Bern, which was approved by the remaining local ethics committees and the Swiss Association of Research Ethics Committees, the authorisation requirement was waived, as the measurement was reclassified as a quality measurement and thus did not fall under the Swiss Human Research Law and within the remit of research ethics committee.

## Results

### Sample

A total of 138 hospitals and 35,998 patients participating in the 2017, 2018 and 2019 measurements were included in the analysis. The overall participation rate was 75.1%. Most of the hospitals analysed (83.3%) were general hospitals. Although university hospitals account for only 3.6% of all hospitals, 19.4% of all patients (*n* = 6,982) came from university hospitals (Table 1).

**Table 1:** Overview of the distribution of the hospitals and patients analysed

Hospitals	Total (*n* = 138)		Patients (*n* = 35,998)	
Hospital types	*n*	%	*n*	%
University hospitals	5	3.6	6,982	19.4
General hospitals	115	83.3	26,590	73.9
Specialised clinics	18	13.0	2,426	6.7

The median age of participants was 70 years and the median length of stay up to measurement was 4 days. Almost half of the patients were female (49.1%, *n* = 17,669). More than three quarters of the patients were either completely care independent (53.5%, *n* = 19,247) or to a great extent care independent (24.5%, *n* = 8,807). About three out of ten patients had fallen in the last 12 months before hospitalization (30.9%, *n* = 11,131) or took sedative or psychotropic medication (35.9%, *n* = 12,928). Altogether, 44.1% (*n* = 15,885) of all participants had undergone a surgical procedure in the 14 days prior to measurement. The three most frequently reported ICD-10 diagnosis groups were diseases of the circulatory system (56.8%, *n* = 20,447), diseases of the musculoskeletal system (40.6%, *n* = 14,626) and endocrine, nutritional and metabolic diseases (35.0%, *n* = 12,617). Further details on patient characteristics can be found in Table 2.

**Table 2:** Overview of participants’ characteristics

Characteristics	Total participants (*n* = 35,998)	
	Median	IQR
Age (years)	70	23
Length of stay up to measurement (days)	4	7
	*n*	% (95% *CI*)
Sex (female)	17,669	49.1 (48.6–49.6)
Care dependency (CDS)		
care independent (70–75)	19,247	53.5 (53.0–54.0)
to a great extent independent (60–69)	8,807	24.5 (24.0–24.9)
partially dependent (45–59)	5,218	14.5 (14.1–14.9)
to a great extent dependent (25–44)	2,083	5.8 (5.5–6.0)
completely dependent (15–24)	643	1.8 (1.7–1.9)
Fall in the last 12 months (yes)	11,131	30.9 (30.4–31.4)
Sedative/psychotropic medications intake (yes)	12,928	35.9 (35.4–36.4)
Surgical procedure within 14 days prior to measurement (yes)	15,885	44.1 (43.6–44.6)
ICD-10—Diseases of the circulatory system	20,447	56.8 (56.3–57.3)
ICD-10—Diseases of the musculoskeletal system and connective tissue	14,626	40.6 (40.1–41.1)
ICD-10—Endocrine, nutritional and metabolic diseases	12,617	35.0 (34.6–35.5)
ICD-10—Diseases of the genitourinary system	11,387	31.6 (31.2–32.1)
ICD-10—Diseases of the digestive system	9,672	26.9 (26.4–27.3)
ICD-10—Diseases of the respiratory system	8,930	24.8 (24.4–25.3)
ICD-10—Neoplasms	7,895	21.9 (21.5–22.4)
ICD-10—Mental, behavioural and neurodevelopmental disorders	6,932	19.3 (18.9–19.7)
ICD-10—Diseases of the blood and blood-forming organs	6,074	16.9 (16.5–17.3)
ICD-10—Diseases of the nervous system	5,172	14.4 (14.0–14.7)
ICD-10—Certain infectious and parasitic diseases	4,788	13.3 (13.0–13.7)
ICD-10—Diseases of the skin and subcutaneous tissue	2,874	8.0 (7.7–8.3)
ICD-10—Factors influencing health status and contact with health services	2,513	7.0 (6.7–7.2)
ICD-10—Injury, poisoning and certain other consequences of external causes	2,377	6.6 (6.4–6.9)
ICD-10—Diseases of the eye and adnexa	2,241	6.2 (6.0–6.5)
ICD-10—Symptoms, signs and abnormal clinical and laboratory findings	1,974	5.5 (5.3–5.7)
ICD-10—Diseases of the ear and mastoid process	959	2.7 (2.5–2.8)
ICD-10—External causes of morbidity	618	1.7 (1.6–1.9)

*IQR* interquartile range, *95% CI* 95% confidence interval, *CDS* Care Dependency Scale, *ICD-10* International Statistical Classification of Diseases and Related Health Problems 10th Revision, the ICD-10 diagnosis groups “Congenital malformations, deformations and chromosomal abnormalities”, “Pregnancy, childbirth and the puerperium” and “Certain conditions originating in the perinatal period” were assigned in less than 1% of the cases and were therefore not included in the analyses

### Inpatient fall rates

In total, 1,239 participants experienced an inpatient fall, corresponding to a fall rate of 3.4% (95% *CI* = 3.3%-3.6%) across all hospitals in Switzerland. When looking at hospital types separately, university hospitals had the highest inpatient fall rates (3.8%, 95% *CI* = 3.3%-4.2%), followed by general hospitals (3.4%, 95% *CI* = 3.2%-3.6%) and specialised clinics (3.2%, 95% *CI* = 2.5%-3.9%). The differences are statistically not significant as the 95% confidence intervals all overlap. The inpatient fall rates per hospital vary between 0.0% and 11.2%. In total, eight hospitals reported no inpatient falls.

### Unadjusted hospital comparison

**Fig. 1:**
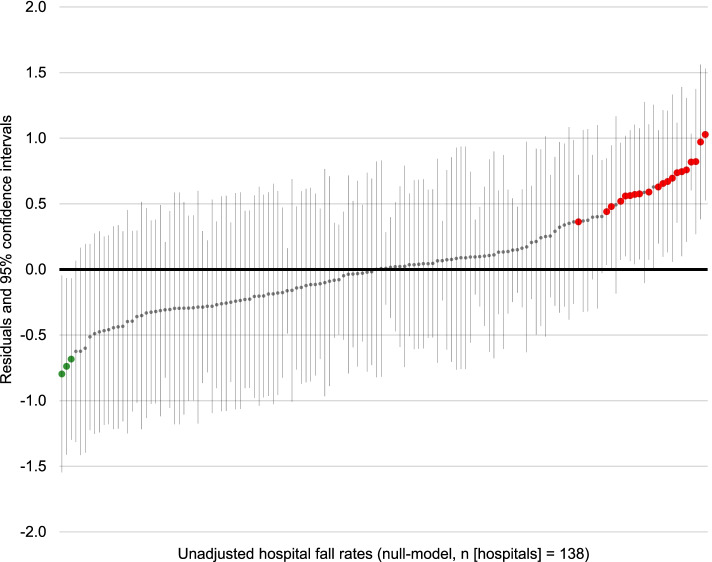
Multilevel unadjusted comparison of hospital inpatient fall rates. For each hospital, the mean residual with its corresponding 95% confidence interval is shown. The horizontal zero line indicates the overall average. Hospitals with 95% confidence intervals not overlapping the zero line are either classified as high-performing hospitals (indicated by green dots) or low-performing hospitals (indicated by red dots) compared with the overall average

Figure 1 presents the multilevel unadjusted hospital inpatient fall rates based on the null-model, i.e. no patient-related fall risk factor covariates are included in this model. Red dots highlight 20 (14.5%) hospitals out of the 138 analysed that had a significantly higher inpatient fall rate compared to the overall average when no risk adjustment was performed (low-performing hospitals). These hospitals were distributed among hospital types as follows: one university hospital, 16 general hospitals and three specialised clinics. In addition, highlighted with green dots, three hospitals (two general hospitals and one specialised clinic) had a lower inpatient fall rate than the overall average (high-performing hospitals).

### Inpatient fall risk adjustment model

The unadjusted and the newly developed inpatient fall risk adjustment model, which includes patient-related fall risk factor covariates, are presented in Table 3 with their corresponding model fit indices. Compared to the unadjusted model, the inpatient fall risk adjustment model showed a significantly better model fit according to the log-likelihood ratio test and the lower Akaike Information Criterion (AIC) value. Furthermore, the conditional *R*^*2*^ shows that the inclusion of fixed effects (patient-related fall risk factor covariates) in the inpatient fall risk adjustment model increases the explained portion of the total variance from 7.1% to 25.8%. The Intraclass Correlation Coefficient (ICC) in the unadjusted model indicates that 7% of inpatient falls can be explained by between-hospital differences and, conversely, 93% by within-hospital differences. After adjusting for patient-related risk factors, the ICC decreased to 3% in the inpatient fall risk model.

**Table 3:** Description and comparison of the unadjusted and inpatient fall risk adjustment model

	**Unadjusted model** **(null model)**			**Inpatient fall** **risk adjustment model**		
*Predictors*	*Odds Ratios*	95% *CI*	*p*	*Odds Ratios*	95% *CI*	*p*
Intercept	0.03	0.03–0.04	** < 0.001**	0.01	0.01–0.01	** < 0.001**
Age [in years]				1.01	1.01–1.02	** < 0.001**
CDS [care independent]				Reference		
CDS [to a great extent independent]				2.16	1.82–2.56	** < 0.001**
CDS [partially dependent]				2.98	2.49–3.57	** < 0.001**
CDS [to a great extent dependent]				3.43	2.78–4.23	** < 0.001**
CDS [completely dependent]				1.87	1.28–2.72	**0.001**
Fall in the last 12 months [yes]				2.14	1.89–2.42	** < 0.001**
Sedative/psychotropic medication intake [yes]				1.74	1.54–1.98	** < 0.001**
ICD-10—Mental and behavioural disorders [yes]				1.55	1.36–1.77	** < 0.001**
ICD-10—Neoplasms [yes]				1.26	1.10–1.44	**0.001**
ICD-10—Diseases of the blood and blood-forming organs [yes]				1.23	1.07–1.41	**0.004**
ICD-10—Certain infectious and parasitic diseases [yes]				1.19	1.02–1.39	**0.024**
ICD-10—Factors influencing health status [yes]				1.21	0.99–1.46	0.058
ICD-10—Diseases of the nervous system [yes]				1.16	1.00–1.34	**0.046**
ICD-10—Endocrine, nutritional and metabolic diseases [yes]				1.13	1.00–1.27	**0.049**
ICD-10—Diseases of the musculoskeletal system [yes]				0.90	0.79–1.02	0.094
Surgical procedure within 14 days prior to measurement [yes]				0.83	0.73–0.95	**0.006**
Sex [female]				0.78	0.70–0.88	** < 0.001**
ICD-10—Diseases of the ear [yes]				0.67	0.47–0.96	**0.030**
**Random Effects**						
σ^2^ [residual variance]	3.29			3.29		
τ_00_ [variability in hospital intercepts]	0.25			0.09		
ICC [Intraclass Correlation Coefficient]	0.07			0.03		
N [hospitals]	138			138		
Observations	35,998			35,998		
Marginal *R*^*2*^ / Conditional *R*^*2*^	0.000 / 0.071			0.239 / 0.258		
AIC [Akaike Information Criterion]	10,684.658			9545.022		
Log likelihood	-5340.329			-4752.511		

^*^Log likelihood ratio test: Chi^2^: *p* < .001, *ICD-10* International Statistical Classification of Diseases and Related Health Problems 10th Revision, *95% CI* 95% confidence interval, *ICC* Intraclass Correlation Coefficient, *CDS* Care Dependency Scale

The inpatient fall risk adjustment model revealed that the following covariates contributed to inpatient fall risk (see also supplementary Fig. 1 for a graphical overview): higher age (Odds Ratio [*OR*] 1.01, 95% *CI* 1.01–1.02, *p* < 0.001), increasing care dependency (*OR* increasing up to the category “to a great extent dependent”, *OR* 3.43, 95% *CI* 2.78–4.23, *p* < 0.001), a fall in the last 12 months (*OR* 2.14, 95% *CI* 1.89–2.42, *p* < 0.001), the intake of sedative and or psychotropic medications (*OR* 1.74, 95% *CI* 1.54–1.98, *p* < 0.001), and the ICD-10 diagnosis groups “Mental and behavioural disorders” (*OR* 1.55, 95% *CI* 1.36–1.77, *p* < 0.001), “Neoplasms” (*OR* 1.26, 95% *CI* 1.10–1.44, *p* = 0.001), “Disease of the blood and blood forming organs” (*OR* 1.23, 95% *CI* 1.07–1.41, *p* = 0.004), “Certain infectious and parasitic diseases” (*OR* 1.19, 95% *CI* 1.02–1.39, *p* = 0.024), “Diseases of the nervous system” (*OR* 1.16, 95% *CI* 1.00–1.34, *p* = 0.046) and “Endocrine, nutritional and metabolic diseases” (*OR* 1.13, 95% *CI* 1.00–1.27, *p* = 0.049).

Two additional ICD-10 diagnosis groups, “Factors influencing health status” and “Diseases of the musculoskeletal system”, were included in the model, but these did not prove to be statistically significant.

The model also showed that some factors reduce the risk of falling and are therefore known as protective factors. The risk of falling appeared to be reduced for females (*OR* 0.78, 95% *CI* 0.70–0.88, *p* < 0.001), patients who have undergone a surgical procedure within 14 days prior to measurement (*OR* 0.83, 95% *CI* 0.73–0.95, *p* = 0.006) and/or patients with “Diseases of the ear” (*OR* 0.67, 95% *CI* 0.47–0.96, *p* = 0.030).

### Risk-adjusted hospital comparison

**Fig. 2:**
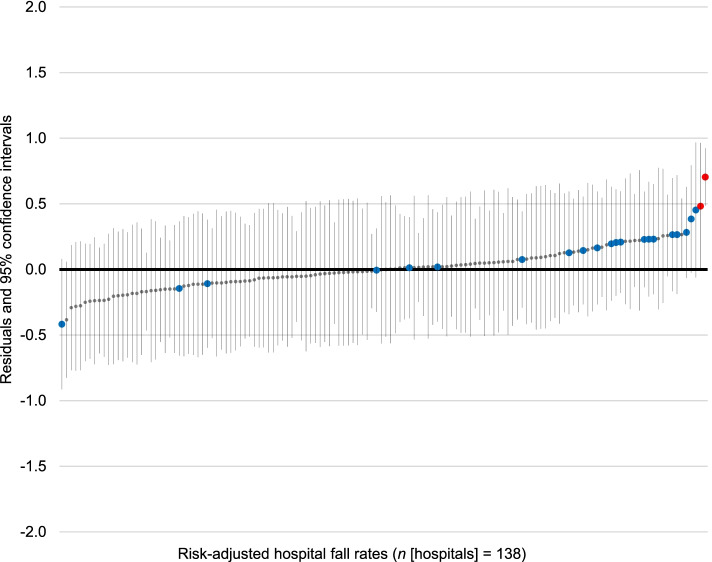
Multilevel risk-adjusted comparison of hospital inpatient fall rates. The red dots indicate hospitals with significantly higher inpatient fall rates compared with the overall average. The blue dots indicate hospitals with significantly higher or lower fall rates in the unadjusted but not in the risk-adjusted comparison

The risk-adjusted comparison of hospitals shows (Fig. 2) that after adjusting for patient-related fall risk factors two hospitals deviate statistically significantly from the overall average. These two hospitals had higher risk-adjusted inpatient fall rates and are therefore categorised as low-performing hospitals when it comes to fall rates. No hospital had a lower risk-adjusted inpatient fall rate (high-performing hospital) than the overall average.

The remaining 21 (91.3%) hospitals that had shown either higher inpatient fall rates (low-performing hospitals) or lower inpatient fall rates (high-performing hospitals) in the unadjusted hospital comparison, in the new model no longer deviated significantly from the overall average in the risk-adjusted hospital comparison.

## Discussion

This article describes the development of a model for risk adjustment of inpatient fall rates in acute care hospitals based on patient-related fall risk factors and presents the impact and results of risk adjustment on hospital performance comparison across Swiss acute care hospitals.

The newly developed risk adjustment model revealed that age, sex, care dependency, fall history, the intake of sedative and or psychotropic medications, surgery and six ICD-10 diagnosis groups are statistically significantly associated with inpatient falls in acute care hospitals in Switzerland. We demonstrated that adjusting for these factors has a relevant impact on the results of hospital performance comparison, as it reduces the number of low as well as high-performing hospitals. In general, it can be stated that the variability of Swiss hospital performance, especially after risk adjustment, was small. Therefore, we can conclude that Swiss hospitals, regardless of hospital type, show a comparable level of care quality with respect to inpatient falls, after adjusting for patient-related fall risk factors.

### Inpatient fall risk adjustment model

One of the most crucial steps in the development of a risk adjustment model is the selection of the variables to be used as independent variables in the model. Since we carried out data-driven statistical variable selection in our model development, it is particularly important to critically review the selected risk variables. In accordance with several studies and guidelines [19, 20, 55–59], older age and a fall in the last 12 months proved to be a relevant patient-related fall risk factor in our risk adjustment model. Care dependency also proved to be a relevant risk factor in our model, as well as in the literature [22, 55]. Our study showed that the risk of falling increases with increasing care dependency compared to the reference category “care independent”, with the exception of the category “completely dependent”, which revealed a lower risk of falling compared to the category “to a great extent dependent”, but still a nearly twofold risk of falling compared to the reference category. One possible explanation is that from a certain level of care dependency, mobility is so severely restricted that locomotion is no longer possible or only possible when accompanied by caregivers, and therefore the risk of falling is lower.

The ICD-10 group diagnoses were important to account for relevant comorbidities in the risk adjustment model. The associations between the ICD-10 diagnosis groups selected in the model and the risk of falling in hospital leave room for interpretation. For example, the literature describes that cognitive impairment is associated with a higher risk of falling [19, 20, 22, 55, 59]. Since dementia is classified in the ICD-10 diagnosis group *Mental, behavioural and neurodevelopmental disorders*, this could be a possible explanation for the selection. This may also be true for the ICD-10 diagnosis group *Neoplasms* as there is evidence that, in addition to the established general patient-related fall risk factors, cognitive impairment, metastases, especially brain metastases, but also comorbidities such as anaemia or fatigue are specific fall risk factors in cancer care [55, 60]. The remaining ICD-10 diagnosis groups selected into the risk adjustment model seem to be important for hospital comparison but are possibly, with odds ratios between 1.23 and 0.90, of less importance for clinical practice.

Additionally, three statistically significant protective factors, i.e., factors that reduce the risk of an inpatient fall, were also selected into the model. The association between a surgical procedure and a reduced fall risk has also been described by Severo, Kuchenbecker [61]. In contrast, there is controversial evidence on the extent to which the female gender is associated with a reduced risk of falling [20–22]. It is also unclear how the ICD-10 diagnosis group *diseases of the ear and mastoid process* is related to a reduced risk of falling. Especially since a recent retrospective cohort analysis based on a large sample size showed that hearing loss is associated with a higher risk of falling [62]. The result in our study might be related to the relatively small number of patients coded with this diagnosis group. This is also reflected in the relatively wide 95% confidence interval of the odds ratio.

Generally, the intake of sedative and psychotropic medication is described as a relevant patient-related fall risk factor [20, 63, 64]. Nevertheless, it is a moot point whether the consideration of this variable in the risk adjustment model is appropriate due to the procedural character of the variable. Hospitals cannot influence the proportion of patients they care for who have already been prescribed sedative or psychotropic medication, but a rigid prescription regime and medication review on admission might directly influence how many patients receive these drugs during hospitalisation. When deciding whether to adjust for sedatives and or psychotropic medications to increase the fairness of the hospital comparison, the temporal relation of when the medications were prescribed, before or after hospital admission, may be of importance.

This applies in principle to all risk factors in the model. For example, even if it is not possible for a hospital to influence the age of its patients, it can introduce targeted preventive measures for older patients to prevent falls and thus indirectly reduce the risk of falls associated with older age. In this context, the risk model is not only important to enable a fair hospital comparison, but it is also of clinical relevance, as it informs health care professionals which patient groups with which characteristics are particularly at risk of falling. Preventive measures can thus be applied in a more targeted manner. At the process level, the assessment of these factors and the initiation of suitable preventive measures by the nursing staff in daily practice is essential to reducing fall rates in acute care hospital.

While risk adjustment is of central importance in providing a fair external benchmark, risk adjustment may also unintentionally mask potential for quality improvement. For example, a hospital that treats many high-risk patients may be considered to be performing well after risk adjustment, even though the unadjusted inpatient fall rate is higher than in other hospitals. However, this is only the case if the measured fall rate is lower than would have been expected based on the many high-risk patients. Therefore, the respective hospital has already taken preventive measures to keep the inpatient fall rates lower than expected. But in the context of internal quality improvement and the suffering that every single fall means for the patient, the question arises whether it is enough to be as good as the other hospitals. At best, despite the more difficult initial situation with the many high-risk patients, it is possible for this hospital to reduce the inpatient fall rate by further optimising the prevention measures. It may be unfair, but hospitals with many high-risk patients always have to do more to achieve the goal of low inpatient fall rates. Therefore, it might be advisable for hospital management and staff not to look at the risk-adjusted results in isolation, but in combination with descriptive results on patients’ risk factors, preventive measures and effective inpatient fall rates. The overall picture should form the basis for discussion and analysis in the team in order to identify potential quality issues and initiate appropriate preventive measures.

### Impact of risk adjustment on hospital comparison

The hospital comparison based on the unadjusted inpatient fall rates revealed 20 low-performing and three high-performing hospitals. In contrast, with the risk-adjusted hospital comparison, it was found that 18 of the 20 hospitals were incorrectly classified as low-performing and that all three of the high-performing hospitals were incorrectly classified. On the other hand, no hospital had been incorrectly classified as an average-performing hospital instead of a low- or high-performing outlier. The study by Danek, Earnest [18], that examined the effect of risk adjustment on the clinical comparison of diabetes-related outcomes showed a comparable effect, as the number of clinics classified as low-performing hospitals decreased significantly after risk adjustment.

In general, the main objective of performance measurements is to provide accurate data to various stakeholders to enable informed decision-making [17]. The non-adjusted hospital comparison as a basis for decision-making would result in some hospitals being ranked better or worse than their actual fall rate performance effectively is. This may have far reaching consequences, especially in health systems where financial reimbursement is directly linked to health outcome measures, as is the case in the US for inpatient falls [65], or if the results are published publicly, which might result in reputation damage for the incorrectly classified low-performing hospitals. In addition to the incorrect classification of low-performing hospitals, our risk adjustment also led to the disappearance of high-performing hospitals. According to Danek, Earnest [18], inaccurate representation of high performance can lead to complacency and have a negative impact on motivation to strive for improvement.

### Hospital comparison of inpatient fall rates in Switzerland

The performance of hospitals regarding fall prevention measures is at a comparable level in Switzerland when patient-related fall risk factors are accounted for. The total variance explained by differences between hospitals is 7% in the null model and 3% in the risk-adjusted model. This shows that the variability in performance of Swiss hospitals is generally low and almost disappears after risk adjustment. Therefore, it is questionable if inpatient falls are an appropriate indicator for hospital performance comparison, as only a small amount of variability is explained on hospital level [66]. This is also an ongoing discussion in other research fields such as hospital readmission rates. Hekkert, Kool [67] reported even smaller ICC values of 0.5% to 2.7% at hospital level for readmission rates after different surgical procedures. The National Quality Forum [3] write in their technical report, unfortunately without giving the actual figures, that the ICC of inpatient falls is higher at ward level than at hospital level. Therefore, another question in connection with the low variability between hospitals is whether the wards rather than the hospitals as a grouping variable are of importance. This is supported by evidence that inpatient fall rates vary significantly by ward types. For example, constantly significantly higher fall rates were reported for medical wards than for surgical wards [68]. A risk-adjusted comparison stratified by department type could be a measure to further improve the comparability of the results. However, one problem in examining and comparing ward performance, as in the present study, is that the low number of patients per ward combined with low inpatient fall rates could make the model estimates inaccurate [39]. Continuous measurements with longer survey periods such as monthly, quarterly, or yearly total number of inpatient falls per patient days or the combination of several measurement dates could address this problem.

At the national level, since the variability always refers to the average of all hospitals, no statement can be made as to whether good or bad quality is achieved in Swiss hospitals regarding inpatient falls in general. It is possible that all hospitals perform well or poorly in a homogeneous way. In order to answer this question, risk-adjusted country comparisons, such as the OECD according to Busse, Klazinga [11] is striving for, must be carried out.

### Strengths and limitations

Our study is based on a large representative sample, as almost all Swiss acute care hospitals participated in the three measurements. Data pooling of the three measurements increased the number of participants per hospital and protected the hospitals to a certain extent from a random result, which would otherwise have been more likely with a small number of cases at only one measurement point. Still, and unfortunately, some small institutions had to be excluded from the analyses. However, this had the positive effect of creating ideal conditions for the multilevel analyses and thus counteracting possible bias in the analyses. An additional strength of the study was the rigorous, well defined and standardised data collection procedure, which was accompanied by instruction meetings and manuals. This is particularly relevant for hospital comparisons, as another reason for the variation in outcome, besides hospital performance, may be differences in the definition and data collection procedure of inpatient falls in hospitals [42].

One limitation to consider is that our data are based on a cross-sectional design and therefore our findings on the association between fall risk factors and inpatient falls are not causal but correlational. Since the risk adjustment model only considers patient-related fall risk factors, it can be assumed that these factors were already present to a certain extent before the patient was admitted to the hospital (e.g., age, gender, fall in the last 12 months) the significance of the temporal relationship is rather negligible. Nevertheless, care should be taken in further fall measurements to take the temporal relation into account if possible.

In general, it should be noted that a risk adjustment model can only take into account measurable and reportable factors [10, 27]. Other measurable patient-related fall risk factors described in the literature are, e.g., impaired mobility or gait instability [19, 22, 55, 64], urinary incontinence or frequency [22, 55, 61, 64, 69] malnutrition [19, 59] or sarcopenia [19, 70]. In our analysis, however, it was not possible to adjust for these factors as they were not collected in our measurements. The impact of the inclusion of these other factors on the accuracy of the risk adjustment model should be further investigated.

It should be noted that inpatient falls can also be influenced by structural factors at the department level, such as environmental (e.g., floors, lighting [55]) or organizational features (e.g., skill mix, nurse staffing ratio [71, 72]). We did not include these factors in our risk adjustment model because that are exactly the factors which are under the control of the hospital and thus differentiate between hospitals. A risk adjustment for structural factors would limit the incentive for hospitals to review and improve them.

## Conclusions

Our study provides compelling evidence for a risk adjustment of inpatient fall rates to enable a fairer, more accurate comparison of hospital performance in terms of care and fall prevention. Because risk adjustment significantly reduced the misclassification of hospital performance, it is recommended to use a risk-adjusted comparison of fall rates as a basis for decision-making instead of a non-adjusted hospital comparison. In addition, for clinical practice, it is recommended that staff consider the patient-related fall risk factors identified in the risk adjustment model, such as care dependency, a history of falling and cognitive impairment in the fall risk assessment in order to initiate appropriate preventive measures.

The risk adjustment model should be further reviewed by considering and testing additional patient-related risk factors, such as impaired mobility, nutritional status, sarcopenia, incontinence, polypharmacy, hearing loss and visual impairment, and applying the risk adjustment model in other contexts (national and international). In addition, it would be important to check whether it would make more sense to consider wards as a grouping unit instead of the hospitals.

## Supplementary Information

**Additional file 1: Figure 1.** Overview of predictors included in the inpatient fall risk adjustment model and their corresponding odds ratios.

## Data Availability

The data that support the findings of this study are available from the Swiss National Association for Quality Development in Hospitals and Clinics (ANQ) but restrictions apply to the availability of these data, which were used under license for the current study, and so are not publicly available. Data are however available from the authors upon reasonable request and with permission of the Swiss National Association for Quality Development in Hospitals and Clinics (ANQ).

## Abbreviations

95% *CI*: 95% Confidence Interval
AIC: Akaike Information Criterion
CDS: Care Dependency Scale
ICC: Intraclass Correlation Coefficient
ICD-10: International Statistical Classification of Diseases and Related Health Problems 10th Revision
IQR: Interquartile Range
LPZ: National Prevalence Measurement of Quality of Care (in Dutch: Landelijke Prevalentiemeting Zorgkwaliteit)
MESH: Medical Subject Headings
OECD: Organisation for Economic Co-operation and Development
OR: Odds Ratio
RNAO: Registered Nurses’ Association of Ontario
UK: United Kingdom
US: United States
