# Supplementary material for: Hospital performance comparison of inpatient fall rates; the impact of risk adjusting for patient-related factors: a multicentre cross-sectional survey
Source: BMC Health Serv Res. 2022 Feb 18;22:225. doi: 10.1186/s12913-022-07638-7 (PMC8857794; doi:10.1186/s12913-022-07638-7)
Supplement: Supplementary file 1 — Additional file 1: Figure 1. Overview of predictors included in the inpatient fall risk adjustment model and their corresponding odds ratios. [file 12913_2022_7638_MOESM1_ESM.docx]

Additional file 1

Supplementary figure 1: Overview of predictors included in the inpatient fall risk adjustment model and their corresponding odds ratios.


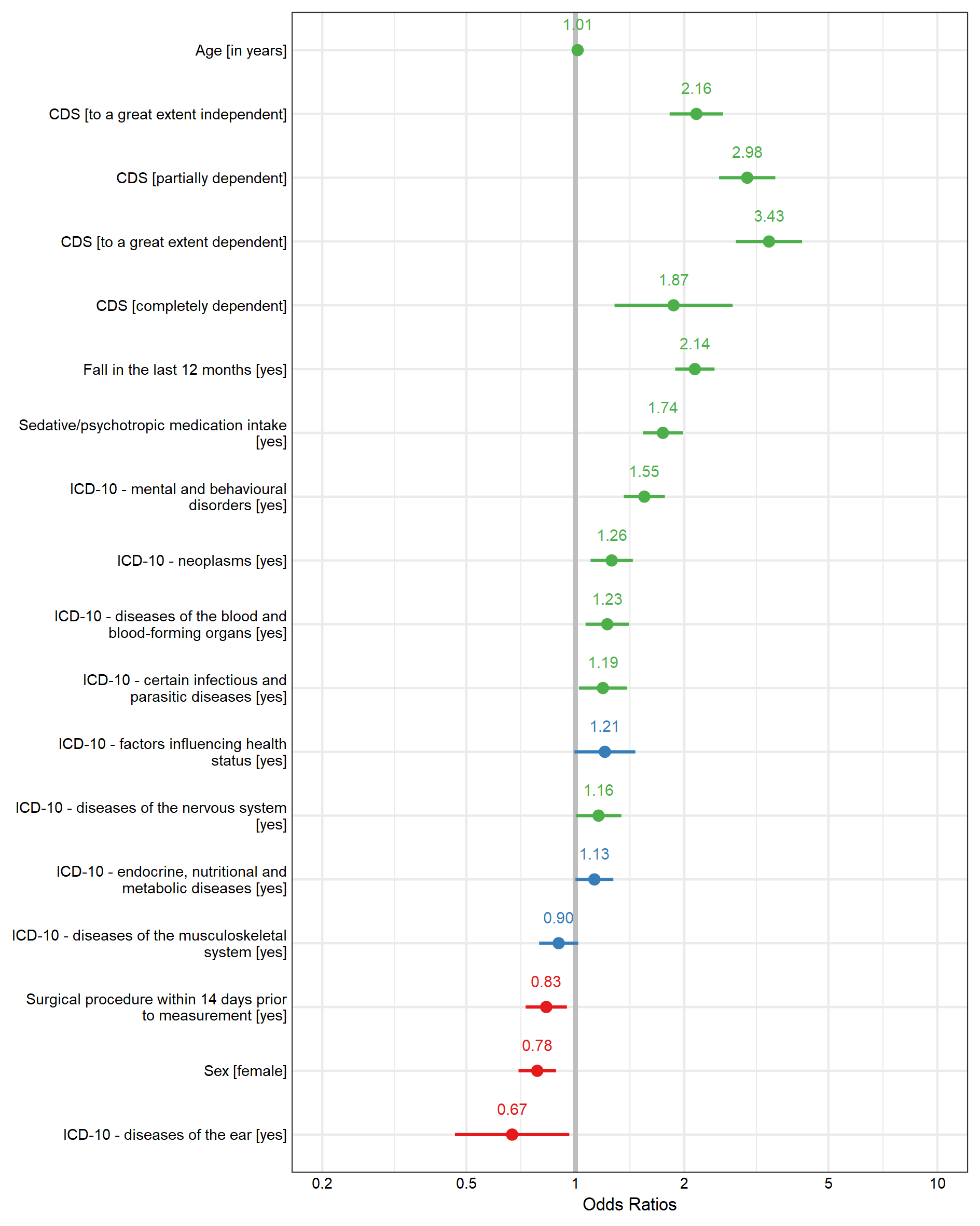


Green values indicate a statistically significant factor increasing inpatient fall risk, blue values indicate a statistically non-significant risk factor and red values indicate a statistically significant factor decreasing inpatient fall risk.
